# A Systemic Pathophysiological View of Sensitive Skin Revealed by Proteomics: Beyond Barrier and Inflammation

**DOI:** 10.1111/jocd.70649

**Published:** 2026-01-07

**Authors:** Lin Guihua, Du Shan, Zhong Xinqing, Lee Yunha, Xiong Zhi

**Affiliations:** ^1^ Amorepacific (Shanghai) D&I Center Co. Ltd Shanghai People's Republic of China

**Keywords:** nonsensitive skin, oxidative stress, proteomics, sensitive skin, skin barrier

## Abstract

**Background:**

Sensitive skin (SS) is a common dermatological condition characterized by enhanced reactivity to environmental, chemical, and cosmetic stimuli, often accompanied by impaired barrier function and discomfort sensation such as burning or itching. Despite its high prevalence, the molecular mechanism underlying SS remains poorly understood.

**Objectives:**

This study aimed to perform a comprehensive proteomics analysis to characterize molecular alterations in SS compared with nonsensitive skin (NS), thereby uncovering key pathways involved in barrier dysfunction, oxidative stress, and neuroinflammatory responses.

**Method:**

Thirty subjects with SS and 30 with NS were recruited. Stratum corneum samples were collected via tape stripping and analyzed using four‐dimensional data‐independent acquisition (DIA) proteomics.

**Results:**

Proteomic profiling revealed distinct molecular signatures between SS and NS. SS exhibited enrichment of pathways related to cytoskeletal remodeling, cell–cell adhesion, and tight junction organization, consistent with impaired but dynamically compensatory barrier regulation. Enhanced oxidative phosphorylation and fatty acid β‐oxidation indicated increased metabolic activity, while elevated glutathione‐related enzyme functions reflected altered redox balance and oxidative stress. Additionally, upregulation of MAPK signaling and neurotrophin‐associated pathways suggested active neuro‐inflammatory crosstalk, potentially contributing to heightened cutaneous sensitivity and inflammatory susceptibility. Additional alterations were observed in other cellular processes, reflecting the complex molecular landscape of SS.

**Conclusion:**

These findings provide new insights into the molecular basis of SS, highlighting the interplay between barrier dysfunction, oxidative stress, and neuronal activation. The identified proteins and pathways may serve as potential biomarkers for SS assessment and as targets for the development of cosmetic products or therapeutic strategies aimed at restoring skin homeostasis.

## 1 Introduction

Sensitive skin (SS) represents a multifaceted condition characterized by heightened cutaneous reactivity to various stimuli. The diagnosis and clinical evaluation of SS syndrome can be effectively supported by a range of specialized sensory testing protocols. These include chemical provocation tests utilizing lactic acid, capsaicin, or dimethyl sulfoxide to assess stinging response; physical challenge methods such as occlusive patch testing and behind‐the‐knee tests; mechanical stimulation procedures, including controlled washing and exaggerated immersion tests [1].

Several studies have suggested a link between SS and a disruption of the epidermal barrier function. SS is known to cause a noticeably decreased amount of neutral lipids and upregulated levels of sphingolipids that have been linked to reduced barrier stability [2]. An impaired skin barrier function compromises its defensive capacity against external stimuli. Studies have shown that exposure to low humidity and cold temperatures can damage the skin barrier function and increase mechanical stress. The molecular mechanisms involve abnormal activation of keratinocytes with subsequent release of pro‐inflammatory cytokines and cortisol, coupled with increased infiltration of dermal mast cells. These pathological changes collectively lead to enhanced cutaneous neurosensitivity and significantly exacerbated immune responses to irritants and allegens [3].

Through genome‐wide association studies (GWAS) comparing SS and nonsensitive skin (NS), researchers have identified that genetic variations affecting oxidative stress responses, cell growth regulation, and neurobiological processes may contribute to skin sensitivity [4]. However, although genomics and lipidomics have provided valuable insights, comprehensive proteomic analyses directly comparing SS and NS remain limited.

Mass spectrometry (MS)‐based proteomics is a method for identifying potential disease biomarkers [5]. The noninvasive tape strip method used in combination with MS proteomics can be used for analysis of skin protein expression in patients with hand eczema [6], atopic dermatitis [7] and aging subjects [8]. Yet, few studies applied these approaches to SS, leaving an important gap in understanding the molecular determinants of this condition.

The present study aims to address this gap by using tape stripping combined with MS‐based proteomic profiling to compare protein expression patterns between SS and NS. We hypothesize that SS will exhibit distinct proteomic signatures related to barrier integrity, inflammatory regulation, oxidative stress, and neurocutaneous signaling. By identifying differentially expressed proteins, this research may provide novel biomarkers for SS and offer mechanistic insights that could guide future therapeutic or cosmetic strategies.

## 2 Methods

### 2.1 Study Subjects and Environment

This study enrolled 30 Chinese subjects with SS and 30 Chinese subjects with NS, aged 25–34. The inclusion criteria for SS subjects were as follows: (1) a moderately positive response in the 10% lactic acid sting test (cumulative score ≥ 5 at 2.5 and 5 min), (2) moderate sensitivity according to the SS questionnaire [4, 9], (3) a self‐reported history of SS persisting for more than 5 years, (4) a trans‐epidermal water loss (TEWL) value > 22 g/m^2^/h on the cheek [10], and (5) presence of facial flushing.

For NS subjects, the criteria were as follows: (1) a negative result in the 10% lactic acid sting test (LAST ‐), (2) TEWL< 17 g/m^2^/h^9^ [10], (3)self‐identification as nonsensitive, and (4)absence of facial flushing.

There is no significant difference in age between the SS and NS subjects (*p* > 0.05). The mean age was 29.80 ± 3.20 years in the SS group and 29.67 ± 3.26 years in the NS group. The SS group comprised 22 females and eight males, while the NS group comprised 26 females and four males.

Subjects with skin diseases at the test site that may affect the evaluation of the trial results, as well as those with a highly allergic constitution, were excluded. Subjects who had recently used medications or cosmetic products that could affect the trial results were excluded. Subjects who maintained irregular lifestyles or had a history of excessive ultraviolet (UV) exposure were also excluded.

Before skin measurements were taken, subjects cleaned their faces and waited 30 min at a controlled temperature of 21°C ± 1°C and a relative humidity of 50% ± 10%.

### 2.2 Protein Extraction, Peptide Enzymatic Hydrolysis, and MS Analysis

SC samples were collected from the cheek using D‐squame tapes. In subjects with SS, samples were taken from the subject‐identified most sensitive area on the cheek, while in NS subjects, samples were taken from the corresponding anatomical region. Each tape was applied with light pressure for 5 s before gentle removal. The first tape strip was discarded to remove surface debris and contaminants. The subsequent three consecutive tape strips were placed in 2 mL grinding tubes and stored at −80°C until further processing.

To each tube, 1 mL of 1% SDC lysis buffer (50 mM Tris–HCl, pH 8.0) and 10 μL of protease inhibitor cocktail (PIC) were added. Samples were subjected to low‐temperature ultrasonication for 40 min, followed by shaking at room temperature for 40 min to extract proteins. Protein concentrations were determined using the BCA assay.

For enzymatic digestion, 50 μL of the lysate from each sample was treated with chloroacetamide (CAA, final concentration 40 mM) and tris (2‐carboxyethyl) phosphine (TCEP, final concentration 10 mM). The mixture was heated at 95°C for 5 min, cooled to room temperature, and digested with trypsin overnight at 37°C. Digestion was quenched with formic acid, and the peptides were desalted using an MCX column. After lyophilization, peptides were reconstituted in 10 μL of 0.1% formic acid, and concentrations were measured at 280 nm. A 600‐ng aliquot was used for MS analysis.

Peptides were separated by nano‐HPLC (Nano Elute system) and analyzed by data‐independent acquisition (DIA) mass spectrometry using a timsTOF instrument (Bruker) in positive ion mode. The MS and MS/MS scanning range was set at 100–1700 m/z. DIA‐PASEF acquisition with 21 windows was applied, and collision energy varied linearly with the ion mobility (1/K0), ranging from 27 to 45 eV corresponding to 1/K0 of 0.85–1.30 Vs/cm^2^.

### 2.3 Measurement of Trans‐Epidermal Water Loss (TEWL)

Skin TEWL was measured at the cheek using a Tewameter (Courage + Khazaka, Köln, Germany). The results were expressed digitally in g/(m^2^·h).

### 2.4 LAST

Lactic acid (purity > 98%, Sigma, USA) was diluted with distilled water to make a concentration of 10%. Drop 50 μL of 10% lactic acid and distilled water, respectively, onto a filter paper with a diameter of 8 mm and randomly place them on the nasolabial folds on both sides of the subject. The subjects evaluated the degree of discomfort such as itching, tingling, and burns at 30 s, 2.5 min, and 5 min, respectively. If the sum of the scores were ≥ 3 on the side of the lactic acid application, the subject was defined as a positive (LAST+); otherwise, as a negative (LAST−).

### 2.5 AMOREPACIFIC Skin Sensitivity Questionnaire

The classification of participants into Sensitive Skin (SS) and Non‐Sensitive Skin (NS) groups was determined using a multi‐step approach. Initially, all participants completed a validated self‐assessment questionnaire for sensitive skin [4, 449]. The final composite sensitive skin score was calculated as (Category A sum × 2) + (Category B sum × 3). Participants with a total score ≥ 10 were preliminarily classified as questionnaire SS, while those with a score < 10 were classified as questionnaire NS (Table 1).

**TABLE 1:** Structure of the sensitive skin assessment questionnaire.

Question items	Answers (Check the box)
Self‐Reported Sensitive Skin	
Do you think your skin is sensitive?	□ No □ Yes
General Skin Status	
1. What is the thickness of your skin?	□ 1. Thin □ 2. Normal □ 3. Thick
2. How does your skin typically change?	□ 1. Rarely changes □ 2. Occasionally changes □ 3. Frequently changes
3. Do you experience skin issues caused by cosmetics?	□ 1. Rarely or Never □ 2. Sometimes □ 3. Often
4. Do you have any skin allergies?	□ 1. No □ 2. Yes, occasionally □ 3. Yes, frequently
5. Do you easily become flushed in the sunlight, or have you experienced sunburn or similar inflammation?	□ 1. No □ 2. Yes, occasionally □ 3. Yes, frequently
Related to Cosmetic Use	
1. After I use alcohol‐rich cosmetics, my skin appears seriously burnt and red.	□ No □ Yes
2. When I apply cosmetics, I experience acne breakouts or skin congestion.	□ No □ Yes
3. When I use cosmetics with strong scent, my skin experiences side effects.	□ No □ Yes
4. When using cosmetics, I always feel itching, heat, burning or other sensations.	□ No □ Yes
5. I can't use cosmetics freely.	□ No □ Yes
6. I only use the cosmetic products that I often use.	□ No □ Yes
7. In the early period of changing cosmetics, I experience mild side effects that disappear with continuous use.	□ No □ Yes
8. I only use soaps that I have regularly applied before.	□ No □ Yes
9. When I change cosmetics, I experience many skin issues.	□ No □ Yes
10. I have never used special cosmetics tailored for sensitive skin.	□ No □ Yes
11. When I used special cosmetics tailored for sensitive skin, I experience side effects.	□ No □ Yes
12. When using cosmetics, my face has become swollen before.	
13. Thick makeup can easily cause acne on my face.	□ No □ Yes
14. I may experience some skin side effects after massage.	□ No □ Yes
15. When removing the facial pack, I might feel red or hot on my face.	□ No □ Yes
Related to Congenital Traits	
1. Have atropic skin.	□ No □ Yes
2. Had the atropic skin in childhood.	□ No □ Yes
3. Somebody in my family has atropine skin.	□ No □ Yes
4. My family comprise individuals who can't use cosmetics freely.	□ No □ Yes
5. Skins of all my family members are comparatively sensitive.	□ No □ Yes
6. The skin is comparatively thin.	□ No □ Yes
7. Blood vessel line are visible on the cheek.	□ No □ Yes
8. Face easily turns red and it is difficult to recover after turning red.	□ No □ Yes
9. Sensitive to metal or jewelry.	□ No □ Yes
10. Sensitive to pollen.	□ No □ Yes
11. Sensitive to food.	□ No □ Yes
12. Suffered from skin measles or dermatitis.	□ No □ Yes
13. If something grows on the skin, it is difficult to disappear.	□ No □ Yes
14. When I am stung or bitten by insects, the swelling become larger than that of others.	□ No □ Yes
Related to Environments	
1. If I am exposed to sunlight, my face becomes red, hot, or itches rapidly.	□ No □ Yes
2. If I am exposed to cold wind, my face turns red.	□ No □ Yes
3. In a dusty area, my face itches and produces unusual substances.	□ No □ Yes
4. My skin changes with weather fluctuations.	□ No □ Yes
5. Temperature differences cause changes in my facial skin.	□ No □ Yes
6. My skin condition is influenced by changes in the surrounding environment.	□ No □ Yes
7. My skin may worsen if the water or soil changes.	□ No □ Yes
8. My skin seems to change with the seasons.	□ No □ Yes
9. If I perspire, my face itches.	□ No □ Yes
Related to Living Habits	
1. Have coprostasis.	□ No □ Yes
2. Often lack sleep.	□ No □ Yes
3. If I have pressure, my skin will often become loose or produce acne.	□ No □ Yes
4. Feel that my skin alters before or after menstruation.	□ No □ Yes
5. After I eat spicy food, my face will produce acne.	□ No □ Yes
6. My skin lacks adaptability.	□ No □ Yes
7. I will take notice of something growing on my face.	□ No □ Yes
8. I feel cool in my hands and feet.	□ No □ Yes
9. Often use ointments on my face.	□ No □ Yes
10. Because I experienced side effects from using ointments, I cannot use them.	□ No □ Yes

*Note:* Scoring is based on two distinct categories. Category A comprises the five items in the “General Skin Status” section, each rated on a 3‐point scale (1–3). Category B encompasses all remaining items (*n* = 48) across the four domains of “Cosmetic Use,” “Congenital Traits,” “Environmental Factors,” and “Living Habits,” each answered in a binary format (No = 0, Yes = 1). This questionnaire is powered by Amorepacific, and all rights are reserved by Amorepacific.

### 2.6 Statistical Analysis

DIA data were processed using Spectronaut software with dynamic iRT retention time prediction, MS2 interference correction, cross‐run normalization, and a maximum of two missed cleavages. Carbamidomethyl (C) was set as a fixed modification, and Oxidation (M) and Acetyl (Protein N‐term) were set as variable modifications. Only results with a *Q* value < 0.01 (FDR < 1%) were retained.

## 3 Results

### 3.1 Differential Protein Expression Analysis Between SS and NS

**FIGURE 1:**
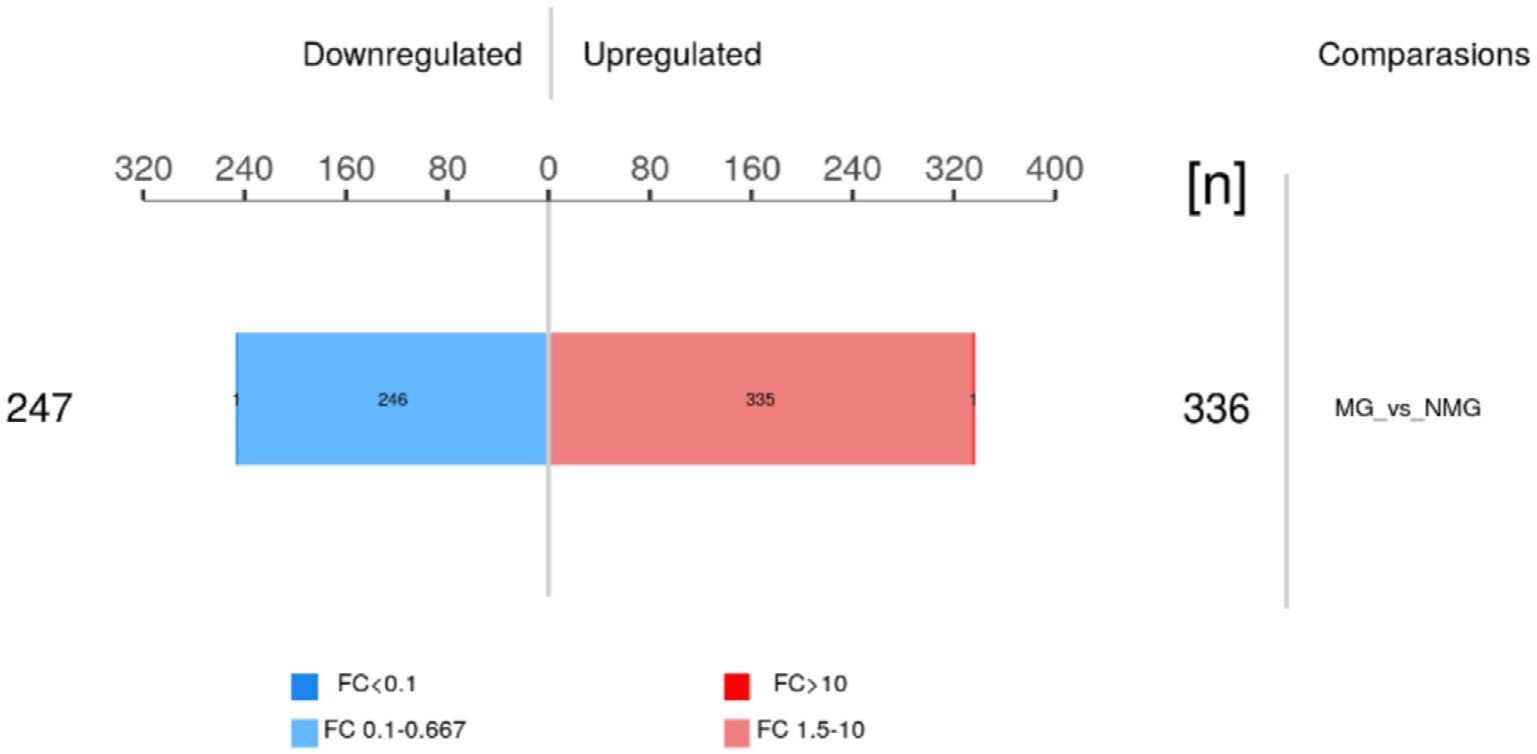
Overview of DEPs between SS and NS groups. Bar plot showing the number of significantly upregulated (red) and downregulated (blue) proteins in SS compared to NS. DEPs were identified based on DIA‐MS analysis using the criteria of fold change > 1.5 or < 0.67 and *p* < 0.05.

Using DIA‐MS (Data‐Independent Acquisition Mass Spectrometry), a total of 4373 proteins were identified from stratum corneum samples. Applying criteria of fold change > 1.5 or < 0.67 and *p* < 0.05, 583 proteins were differentially expressed between the SS and NS, including 336 upregulated and 247 downregulated proteins (Figure 1). Among the top upregulated proteins in SS were ACTG1 (barrier‐related [11]), DYNLT1 and SERPINA4 (inflammation [12] and oxidative stress‐related [1, 13]), and RPA2 (DNA damage and repair‐related [14, 15]) (Table 2). The top downregulated proteins included KPRP, ALOXE3, and PKP1 (barrier‐related [16, 17, 18, 19]), YIPF2 (DNA damage response‐related [20]), GNB2 (inflammation‐related [21]), and KRT9 (epidermal differentiation‐related [22]) (Table 3).

**TABLE 2:** Top 10 upregulated proteins in SS group. List of the top 10 upregulated proteins in SS compared to NS, ranked by fold change. Protein names, gene names, fold change values, and statistical significance are shown.

Protein name	Gene name	Fold change SS/NS	*p* value	Upregulated or downregulated
P63261	ACTG1	18.19	3.43543E‐15	Up
P63172	DYNLT1	3.06	4.96267E‐13	Up
P29622	SERPINA4	2.83	1.25632E‐13	Up
P15927	RPA2	2.70	1.59023E‐12	Up
Q99417	MYCBP	2.17	2.07091E‐12	Up
Q9NQP4	PFDN4	2.26	1.07895E‐11	Up
Q9Y5J7	TIMM9	2.93	1.67157E‐11	Up
Q9UJC5	SH3BGRL2	2.70	2.59311E‐11	Up
P21964	COMT	2.21	2.1246E‐11	Up
Q9Y333	LSM2	1.70	2.41221E‐10	Up

**TABLE 3:** Top 10 downregulated proteins in SS group.

Protein name	Gene name	Fold change SS/NS	*p*	Upregulated or downregulated
Q9NQ36	SCUBE2	0.12	6.21146E‐09	Down
Q5T749	KPRP	0.29	5.13079E‐08	Down
Q9BWQ6	YIPF2	0.39	1.40955E‐08	Down
Q9BYJ1	ALOXE3	0.37	8.26628E‐08	Down
P62873	GNB1	0.60	5.96959E‐08	Down
P62879	GNB2	0.62	2.30808E‐07	Down
Q13835	PKP1	0.52	3.80992E‐07	Down
P63092	GNAS	0.46	3.39626E‐07	Down
P35527	KRT9	0.22	2.12404E‐07	Down
Q12802	AKAP13	0.38	1.37623E‐07	Down

*Note:* List of the top 10 downregulated proteins in SS compared to NS, ranked by fold change. Protein names, gene names, fold change values, and statistical significance are shown.

### 3.2 Gene Ontology (GO) Functional Enrichment

GO enrichment analysis revealed that DEPs were predominantly associated with epidermal structure, cytoskeleton, metabolism, and redox regulation.

**FIGURE 2:**
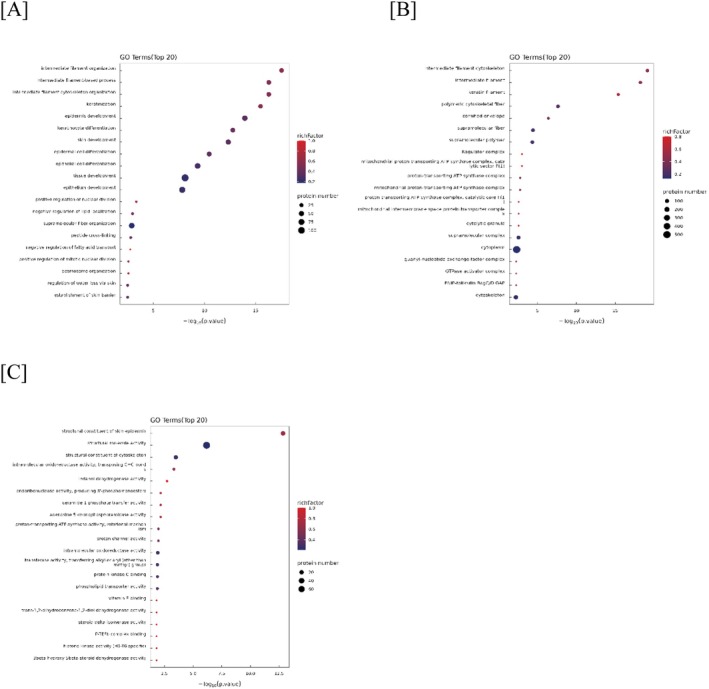
Gene ontology (GO) enrichment analysis of differentially expressed proteins in SS compared with NS. Top 20 significantly enriched GO biological processes (BP). Top 20 significantly enriched GO cellular component (CC). Top 20 significantly enriched GO molecular function (MF). The x‐axis represents the enrichment significance of each GO term, calculated using Fisher's Exact Test and expressed as ‐log10 (*p* value), with higher values indicating greater significance. The bubble color denotes the Rich Factor (the ratio of differentially expressed proteins annotated to a GO term to the total proteins associated with that term), where a shift toward red reflects higher values. Bubble size corresponds to the number of differentially expressed proteins within each GO term.

GO biological process (BP): Among the DEPs, GO biological process enrichment revealed significant involvement in intermediate filament organization, keratinization, epidermal development, and keratinocyte differentiation (Figure 2A). Terms related to skin barrier formation, including “regulation of water loss via skin” and “establishment of skin barrier”, were also significantly enriched. These results suggest that alterations in the structural and differentiation pathways of keratinocytes are closely associated with SS.

GO cellular component (CC): Altered proteins localized mainly to intermediate filament cytoskeleton, keratin filaments, cornified envelope, and mitochondrial ATP synthase complexes, highlighting structural and metabolic involvement (Figure 2B).

GO molecular function (MF): Proteins with structural molecule activity, cytoskeletal binding, oxidoreductase activity, proton channel function, and lipid/ceramide transport were enriched, suggesting metabolic and redox regulation differences in SS (Figure 2C).

### 3.3 Kyoto Encyclopedia of Genes and Genomes (KEGG) Pathway Enrichment Analysis

**FIGURE 3:**
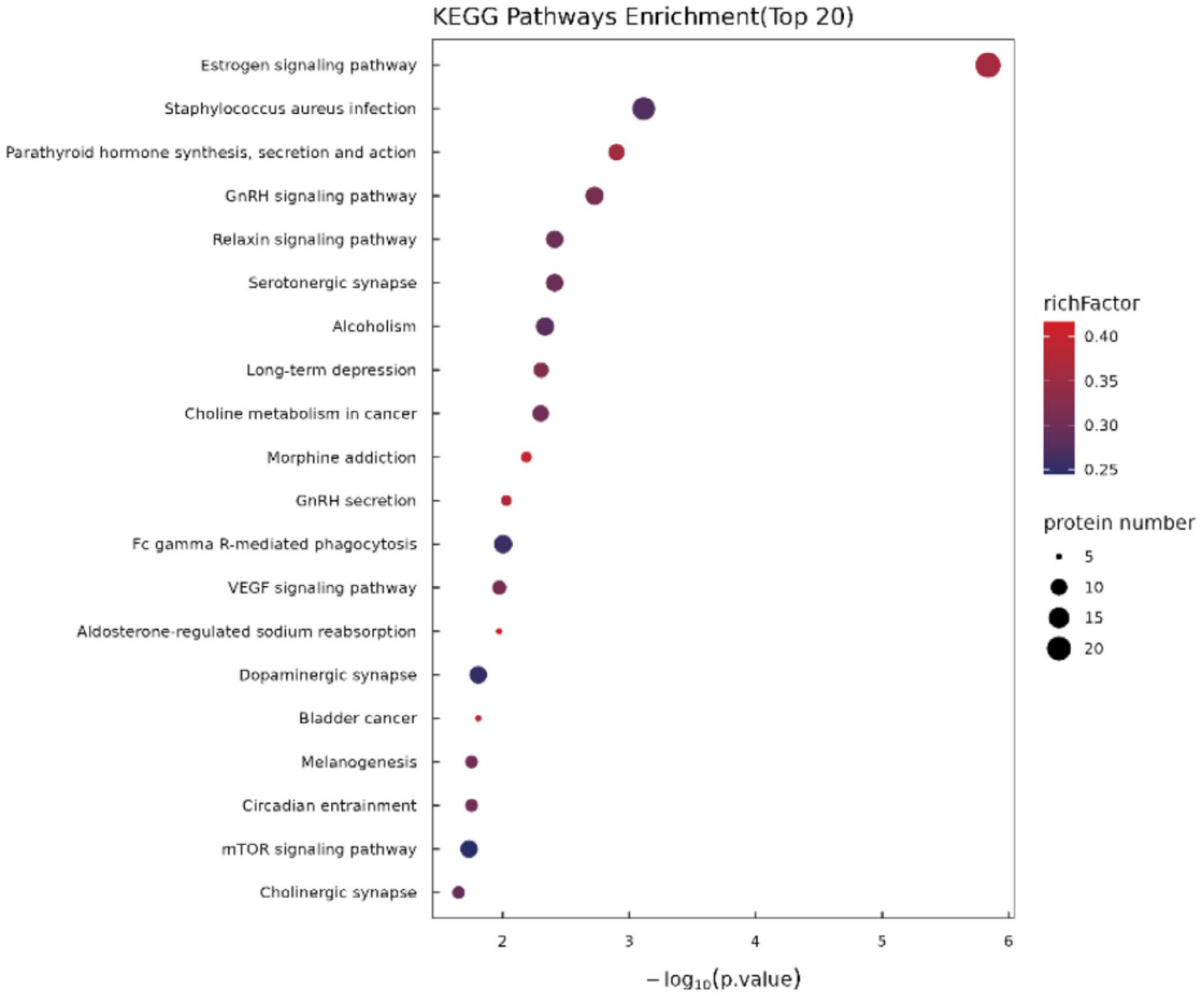
KEGG pathway enrichment analysis (Top 20). The x‐axis represents the enrichment significance of each KEGG pathway, calculated using Fisher's Exact Test (−log_10_ *p* value). A larger x‐axis value indicates a higher level of enrichment significance. The color gradient represents the rich factor, defined as the ratio of differentially expressed proteins annotated to a given KEGG pathway to the total number of identified proteins in that pathway; colors closer to red indicate larger rich factor values. The size of each bubble reflects the number of differentially expressed proteins annotated to the corresponding KEGG pathway.

KEGG analysis identified the top 20 significantly enriched pathways in SS, including estrogen signaling, immune response (e.g., *Staphylococcus aureus* infection, Fc gamma‐R‐mediated phagocytosis), hormone regulation (GnRH, relaxin, and parathyroid hormone), synaptic pathways (serotonergic, dopaminergic, cholinergic), VEGF signaling, mTOR signaling, and cancer‐related pathways (choline metabolism, bladder cancer) (Figure 3). These results indicate broad alterations in hormonal, neural, immune, and cell‐growth‐related pathways in SS.

### 3.4 Skin Barrier

**FIGURE 4:**
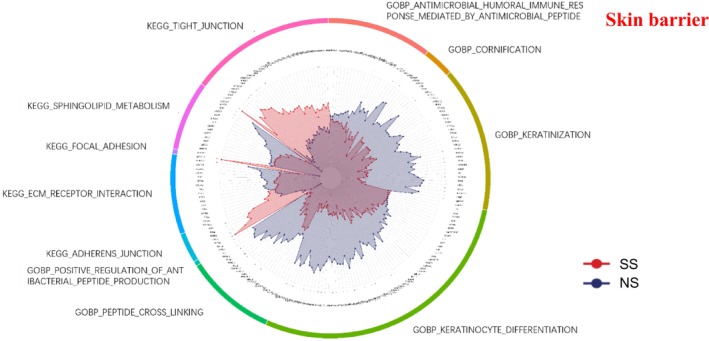
Skin barrier‐related GO terms and KEGG pathways in SS and NS. Radar chart depicting enriched GO and KEGG pathways associated with the skin barrier. Red shading represents SS and blue shading represents NS. The distance from the center of the circle to the data point on each axis represents the average expression value of that biomarker, measured on a *Z*‐score scale. *FDR < 0.05; **FDR < 0.01; ***FDR < 0.001.

SS exhibited widespread downregulation of barrier‐related processes compared with NS (Figure 4). GO biological processes, including keratinization, keratinocyte differentiation, cornification, peptide cross‐linking, and antimicrobial defense, were reduced. In contrast, KEGG tight junction and adherens junction pathways were upregulated in SS.

### 3.5 Cytoskeleton, Adhesion, and Junction

**FIGURE 5:**
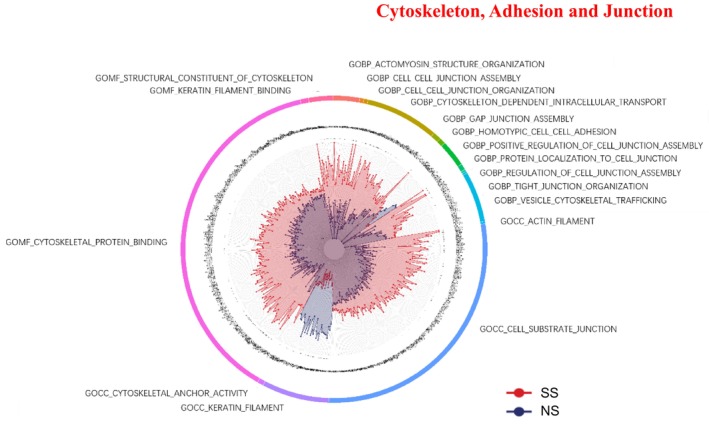
Cytoskeleton, adhesion, and junction‐related GO terms and KEGG pathways in SS and NS. Radar chart depicting enriched GO and KEGG pathways associated with the cytoskeleton, adhesion, and junction. Red shading represents SS and blue shading represents NS. The distance from the center of the circle to the data point on each axis represents the average expression value of that biomarker, measured on a *Z*‐score scale. *FDR < 0.05; **FDR < 0.01; ***FDR < 0.001.

Cytoskeletal and junction‐related GO terms were generally enriched in SS (Figure 5). Proteins involved in cytoskeletal protein binding, structural constituents of cytoskeleton, and keratin filament binding were elevated in SS. Adhesion‐ and junction‐related processes, including cell–cell junction assembly, regulation of junctions, homotypic cell adhesion, and tight junction organization, were also upregulated in SS. Additionally, cytoskeleton‐dependent intracellular transport and vesicle trafficking pathways were enriched in SS. At the cellular component level, actin filaments, cytoskeletal anchors, and cell–substrate junctions were enriched in SS, whereas keratin filaments were relatively downregulated.

### 3.6 Energy Metabolism

**FIGURE 6:**
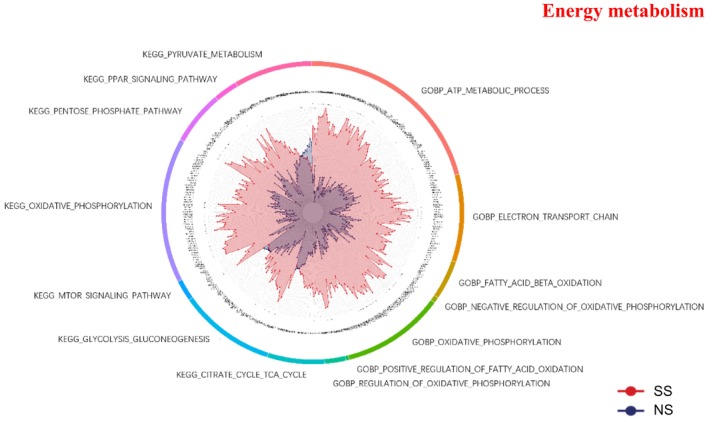
Energy metabolism‐related GO terms and KEGG pathways in SS and NS. Radar chart depicting enriched GO and KEGG pathways associated with energy metabolism. Red shading represents SS and blue shading represents NS. The distance from the center of the circle to the data point on each axis represents the average expression value of that biomarker, measured on a *Z*‐score scale. *FDR < 0.05; **FDR < 0.01; ***FDR < 0.001.

SS displayed broad metabolic activation compared with NS (Figure 6). KEGG analysis revealed significant enrichment of oxidative phosphorylation, while GOBP terms such as fatty acid β‐oxidation and electron transport chain were also elevated. Regulatory processes, including the regulation of oxidative phosphorylation and positive regulation of fatty acid oxidation were enriched in SS.

### 3.7 Oxidative Stress

**FIGURE 7:**
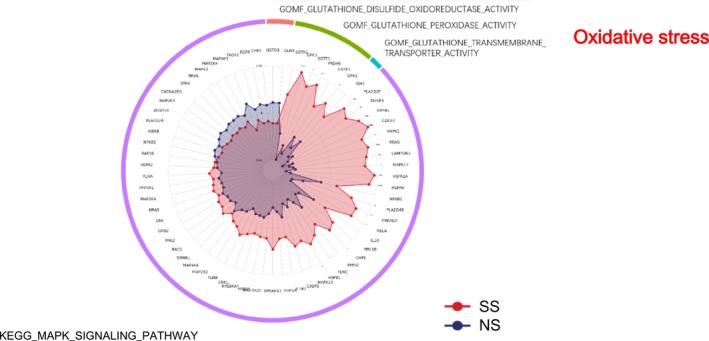
Oxidative stress‐related GO terms and KEGG pathways in SS and NS. Radar chart depicts enriched GO and KEGG pathways associated with oxidative stress. Red shading represents SS and blue shading represents NS. The distance from the center of the circle to the data point on each axis represents the average expression value of that biomarker, measured on a *Z*‐score scale. *FDR < 0.05; **FDR < 0.01; ***FDR < 0.001.

GO molecular function analysis indicated altered redox regulation in SS (Figure 7). Glutathione disulfide oxidoreductase activity, glutathione transporter activity, and glutathione peroxidase activity were all elevated in SS. KEGG pathway analysis also showed upregulation of the MAPK signaling pathway.

### 3.8 DNA Damage Repair

**FIGURE 8:**
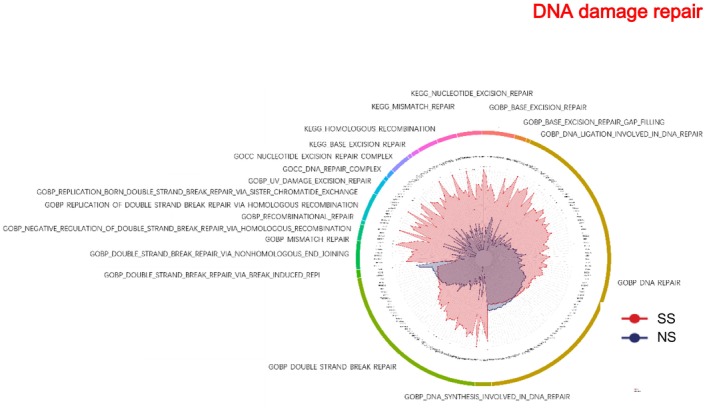
DNA damage repair‐related GO terms and KEGG pathways in SS and NS. Radar chart depicting enriched GO and KEGG pathways associated with DNA damage repair. Red shading represents SS and blue shading represents NS. The distance from the center of the circle to the data point on each axis represents the average expression value of that biomarker, measured on a *Z*‐score scale. *FDR < 0.05; **FDR < 0.01; ***FDR < 0.001.

SS exhibited higher enrichment across a broad range of DNA repair processes compared with NS (Figure 8). In the GOBP category, SS was upregulated in DNA repair, double‐strand break repair, homologous recombination, nonhomologous end joining, and mismatch repair. Cellular components such as nucleotide excision repair and DNA repair complexes were enriched. KEGG pathways, including nucleotide excision repair, base excision repair, and mismatch repair, were also more enriched in SS.

### 3.9 Skin Inflammation

**FIGURE 9:**
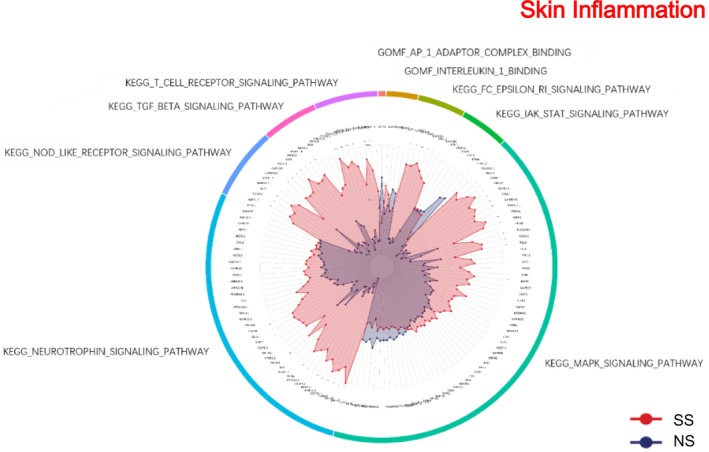
Skin inflammation‐related GO terms and KEGG pathways in SS and NS. Radar chart depicting enriched GO and KEGG pathways associated with skin inflammation. Red shading represents SS and blue shading represents NS. The distance from the center of the circle to the data point on each axis represents the average expression value of that biomarker, measured on a *Z*‐score scale. *FDR < 0.05; **FDR < 0.01; ***FDR < 0.001.

Inflammatory signaling pathways were broadly upregulated in SS (Figure 9). KEGG analysis highlighted JAK–STAT, MAPK, and T cell receptor, NOD‐like receptor, and TGF‐β signaling. Molecular function terms such as interleukin‐1 binding and AP‐1 adaptor complex binding were also enriched, reflecting a pro‐inflammatory cytokine profile.

### 3.10 Neuronal Signaling and Receptors

**FIGURE 10:**
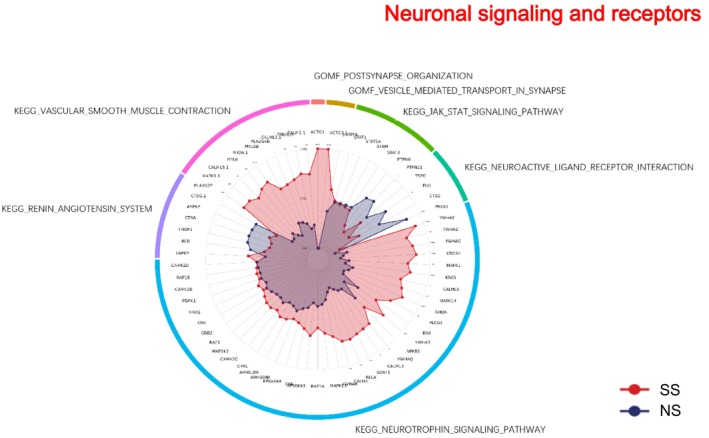
Neuronal signaling and receptors—related GO terms and KEGG pathways in SS and NS. Radar chart depicting enriched GO and KEGG pathways associated with neuronal signaling and receptors. Red shading represents SS and blue shading represents NS. The distance from the center of the circle to the data point on each axis represents the average expression value of that biomarker, measured on a *Z*‐score scale. *FDR < 0.05; **FDR < 0.01; ***FDR < 0.001.

SS showed broad upregulation of neuronal and inflammatory signaling pathways (Figure 10). KEGG neuroactive ligand receptor interaction pathway was downregulated. In contrast, KEGG neurotrophin signaling and KEGG vascular smooth muscle contraction were upregulated. GOMF terms, including postsynapse organization and vesicle‐mediated transport in synapse, were also enriched.

### 3.11 Lipid Metabolism

**FIGURE 11:**
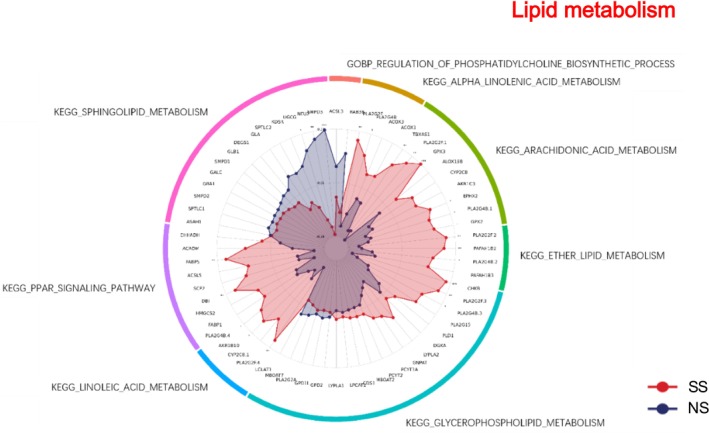
Lipid metabolism‐related GO terms and KEGG pathways in SS and NS. Radar chart depicting enriched GO and KEGG pathways associated with lipid metabolism. Red shading represents SS and blue shading represents NS. The distance from the center of the circle to the data point on each axis represents the average expression value of that biomarker, measured on a *Z*‐score scale. *FDR < 0.05; **FDR < 0.01; ***FDR < 0.001.

Lipid metabolism pathways showed distinct enrichment patterns between SS and NS (Figure 11). In SS, KEGG arachidonic acid metabolism, KEGG ether lipid metabolism, KEGG glycerophospholipid metabolism, KEGG linoleic acid metabolism, KEGG alpha linolenic acid metabolism, and KEGG PPAR signaling pathway were upregulated, whereas KEGG sphingolipid metabolism was downregulated.

### 3.12 Skin Pigmentation

**FIGURE 12:**
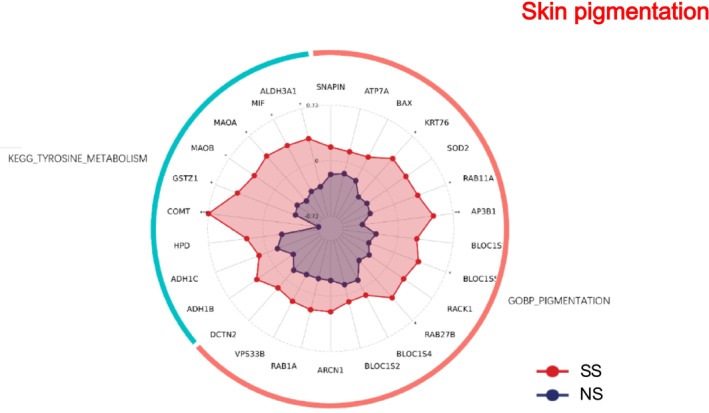
Skin pigmentation‐related GO terms and KEGG pathways in SS and NS. Radar chart depicting enriched GO and KEGG pathways associated with skin pigmentation. Red shading represents SS and blue shading represents NS. The distance from the center of the circle to the data point on each axis represents the average expression value of that biomarker, measured on a Z‐score scale. *FDR < 0.05; **FDR < 0.01; ***FDR < 0.001.

Pigmentation‐related pathways were enriched in SS (Figure 12). Specifically, both the tyrosine metabolism pathway (KEGG) and pigmentation‐related biological processes were upregulated.

## 4 Discussion

In this study, we conducted a comprehensive proteomic analysis to elucidate the molecular features distinguishing SS from NS. Our results demonstrate that SS is characterized by a constellation of molecular alterations, including impaired barrier function, enhanced cytoskeletal and junctional remodeling, metabolic activation and oxidative stress, DNA repair activation, inflammatory and neuroimmune signaling alterations, and dysregulated lipid metabolism—all collectively contributing to heightened cutaneous reactivity. The identification of differentially expressed proteins, along with enriched KEGG pathways and GO functions, provides not only mechanistic insight but also reveals potential biomarkers for SS.

### 4.1 Impaired Skin Barrier

SS exhibited a widespread downregulation of barrier‐related processes compared to NS. Key GO biological processes—including keratinization, cornification, keratinocyte differentiation, and antimicrobial defense—were significantly suppressed. Notable among the top downregulated proteins were KPRP, ALOXE3, PKP1, and KRT9, underscoring a deficit in epidermal differentiation and structural integrity [23, 24]. In contrast, KEGG pathways associated with tight and adherens junctions were upregulated, potentially reflecting a compensatory mechanism to preserve residual barrier function [25, 26]. The significant enrichment of GO terms related to “regulation of water loss via skin” and “establishment of skin barrier” further affirms the central role of keratinocyte structural and differentiation pathways in SS pathophysiology.

### 4.2 Cytoskeletal and Junctional Remodeling

Our findings establish cytoskeletal and junctional remodeling as pivotal molecular features of SS. The enrichment of actin filaments, cytoskeletal anchors, and biological processes such as cell–cell junction assembly and vesicle‐cytoskeletal trafficking points to a compensatory reinforcement of adhesion and intracellular transport dynamics. This is consistent with established evidence that cytoskeletal regulation modulates both epidermal and immune functions [27, 28, 29]. Concurrently, the downregulation of keratin filaments highlights an underlying impairment in keratinocyte differentiation and barrier resilience [30]. This dual pattern suggests that SS involves a simultaneous, and perhaps counteracting, process of compensatory strengthening in cytoskeletal and junctional networks alongside persistent structural vulnerabilities within the epidermis, emphasizing the critical role of integrated adhesion‐cytoskeleton dynamics in maintaining barrier integrity.

### 4.3 Metabolic Activation and Oxidative Stress

SS displayed a broad upregulation of energy metabolism pathways, including oxidative phosphorylation, fatty acid β‐oxidation, and electron transport chain activity. This was accompanied by enhanced glutathione‐related redox activity [31] and MAPK signaling. Proteins such as DYNLT1 and SERPINA4, which are implicated in inflammation and oxidative stress regulation, were among the most significantly upregulated, corroborating these functional observations. These metabolic alterations likely represent adaptive responses to barrier impairment, as mitochondrial ATP production is indispensable for keratinocyte proliferation, repair, and regeneration [32, 33]. Environmental stressors like particulate matter 2.5 (PM2.5) are known to induce ROS accumulation and trigger inflammatory pathways—such as MAPK, COX2/PGE2, and TNF‐α/filaggrin signaling—thereby compromising barrier integrity and promoting inflammation [34]. Given that barrier dysfunction is a hallmark of SS, these metabolic and redox adaptations might initially serve as short‐term protective mechanisms. However, persistent oxidative stress and sustained MAPK activation could ultimately aggravate barrier fragility, thereby contributing to the heightened reactivity characteristic of SS.

### 4.4 DNA Damage Repair

SS showed significantly higher enrichment across a broad range of DNA repair processes compared to NS. Key GO biological processes—including DNA repair, double‐strand break repair, homologous recombination, nonhomologous end joining, and mismatch repair—were consistently upregulated. Cellular components such as nucleotide excision repair complexes and DNA repair complexes were also enriched, while relevant KEGG pathways, including nucleotide excision repair, base excision repair, and mismatch repair, were more prominent in SS. Among the top upregulated proteins, RPA2—a key regulator of DNA replication and repair—exemplifies the enhanced DNA damage response in SS. These findings suggest that heightened DNA repair activity may be a reflection of chronic oxidative and environmental stress in SS, potentially contributing to barrier fragility and epidermal hyper‐reactivity [35].

### 4.5 Inflammation and Neuroimmune Signaling

SS exhibited a broad upregulation of inflammatory pathways. KEGG analysis revealed activation of JAK–STAT, MAPK, T cell receptor, NOD‐like receptor, and TGF‐β signaling. Concurrently, molecular functions such as interleukin‐1 binding and AP‐1 adaptor complex binding were enriched, reflecting a pro‐inflammatory cytokine environment. Neuronal receptor–related pathways demonstrated mixed regulation: while KEGG neuroactive ligand–receptor interactions were downregulated, neurotrophin signaling and vascular smooth muscle contraction were upregulated. Enriched GO terms related to postsynapse organization and vesicle‐mediated transport in synapses suggest underlying neural adaptations that may contribute to altered sensory perception, pain, and itch in SS. Among the differentially expressed proteins, the downregulation of GNB2, involved in inflammatory signal transduction, indicates a complex regulatory landscape for inflammatory mediators in SS. These findings are consistent with previous studies that link barrier dysfunction and neuroimmune signaling to skin inflammation and sensory abnormalities [36, 37, 38, 39].

### 4.6 Lipid Metabolism and Pigmentation

SS demonstrated distinct alterations in lipid metabolism. Several KEGG pathways—including arachidonic acid, ether lipid metabolism, glycerophospholipid metabolism, linoleic acid metabolism, α‐linolenic acid metabolism, and PPAR signaling—were upregulated, whereas sphingolipid metabolism was downregulated. These shifts suggest a substantial reorganization of lipid composition that could influence both barrier properties and inflammatory responses [40, 41, 42, 43].

Pigmentation‐related pathways, notably tyrosine metabolism, were also elevated, indicating that melanogenesis may contribute to physiological differences in SS. This observation aligns with prior studies demonstrating that pigmentation modulates skin sensitivity through tyrosinase‐dependent signaling [44].

### 4.7 Integrated Pathophysiological Model

The findings of this study collectively point toward a self‐perpetuating pathophysiological cycle that may underlie the hyper‐reactivity characteristic of sensitive skin. We propose that a primary disruption in barrier integrity, evidenced by the downregulation of cornified envelope proteins and differentiation markers, serves as the initiating event. A compromised stratum corneum facilitates heightened exposure of cutaneous sensory nerve endings and epidermal cells to environmental stressors, leading to reactive oxygen species accumulation and consequent DNA damage—a notion strongly supported by the observed widespread activation of DNA repair pathways and upregulation of oxidative stress markers. In response, keratinocytes undergo compensatory metabolic reprogramming and cytoskeletal remodeling in an attempt to restore homeostasis and reinforce cellular architecture. However, this state of persistent cellular stress promotes the release of alarmins and cytokines, which in turn activate the cutaneous neuro‐immune axis, ultimately manifesting as the characteristic neuro‐sensory symptoms of stinging, burning, pruritus, and flushing. Therefore, sensitive skin may be redefined as a dynamic, self‐reinforcing cycle, initiated by barrier deficiency and sustained through ongoing cellular stress, dysregulated repair mechanisms, and aberrant neuro‐immune crosstalk.

### 4.8 Study Limitations

This study has several limitations. First, it was conducted at a single center with participants from a single ethnic background (Chinese adults) and within a relatively narrow age range, which may limit the generalizability of the findings to broader populations. Second, all biophysical measurements and proteomic analyses were restricted to a single facial site (cheek), which may not fully capture regional variations across different anatomical sites. Third, the study did not include an external or independent validation cohort, which limits the ability to confirm the reproducibility and robustness of the identified proteomic signatures. Future studies incorporating multi‐center, multi‐ethnic cohorts with wider age ranges, multiple anatomical regions, and external validation will be important to enhance the generalizability of these findings.

## 5 Conclusion

In summary, our proteomic analysis demonstrates that SS is defined by a combination of impaired barrier formation, enhanced cytoskeletal and junctional dynamics, metabolic and oxidative stress, and amplified neuroimmune signaling. These molecular alterations converge to create a state of heightened cutaneous reactivity, providing mechanistic insight into the pathophysiology of SS. Importantly, the identified differentially expressed proteins and pathways may serve as potential biomarkers or therapeutic targets, offering new opportunities for objective diagnosis and tailored intervention. Future studies integrating multi‐omics approaches and functional validation are warranted to further clarify causal mechanisms and to translate these findings into clinical applications.

## Author Contributions

Lin Guihua, Xiong Zhi, Lee Yunha: conceptualization. Lin Guihua, Du Shan, Zhong Xinqing: methodology and data curation. Lin Guihua: writing – original draft. Lin Guihua, Xiong Zhi: writing – review and editing. Xiong Zhi, Lee Yunha: supervision.

## Ethics Statement

The study was conducted in accordance with the Declaration of Helsinki and good clinical practice guidelines. Ethical approval was obtained from the Shanghai Ethics Committee for Clinical Research (Approval No. SECCR/2024‐258‐01).

## Consent

Written informed consent was obtained from all participants prior to their inclusion in the study.

## Conflicts of Interest

The authors declare no conflicts of interest.

## Data Availability

The data that support the findings of this study are available from the corresponding author upon reasonable request.

## References

[1] E. Berardesca , M. Farage , and H. Maibach , “Sensitive Skin: An Overview,” International Journal of Cosmetic Science 35 (2013): 2–8. 22928591 10.1111/j.1468-2494.2012.00754.x

[2] A. B. Cua , K. P. Wilhelm , and H. I. Maibach , “Cutaneous Sodium Lauryl Sulphate Irritation Potential: Age and Regional Variability,” British Journal of Dermatology 123 (1990): 607–613. 2248890 10.1111/j.1365-2133.1990.tb01477.x

[3] K. A. Engebretsen , J. D. Johansen , S. Kezic , A. Linneberg , and J. P. Thyssen , “The Effect of Environmental Humidity and Temperature on Skin Barrier Function and Dermatitis,” Journal of the European Academy of Dermatology and Venereology 30, no. 2 (2016): 223–249. 26449379 10.1111/jdv.13301

[4] S. Y. Kim , K. W. Hong , M. Y. Oh , et al., “Genetic Variants Associated With Sensitive Skin: A Genome‐Wide Association Study in Korean Women,” Life (Basel) 14, no. 11 (2024): 1352. 39598151 10.3390/life14111352 PMC11595562

[5] E. S. Nakayasu , M. Gritsenko , P. D. Piehowski , et al., “Tutorial: Best Practices and Considerations for Mass‐Spectrometry‐Based Protein Biomarker Discovery and Validation,” Nature Protocols 16 (2021): 3737–3760. 34244696 10.1038/s41596-021-00566-6 PMC8830262

[6] J. B. K. Sølberg , A. S. Quaade , L. Drici , et al., “The Proteome of Hand Eczema Assessed by Tape Stripping,” Journal of Investigative Dermatology 143, no. 8 (2023): 1559–1568.e5. 36773646 10.1016/j.jid.2022.12.024

[7] J. W. Chang , X. B. Huang , W. J. Jiang , et al., “Abrocitinib Versus Dupilumab: Impact on Skin Barrier Function and Proteomics in Atopic Dermatitis,” Journal of the American Academy of Dermatology 93, no. 2 (2025): 406–414. 40246083 10.1016/j.jaad.2025.04.027

[8] J. Ma , M. T. Liu , Y. C. Wang , et al., “Quantitative Proteomics Analysis of Young and Elderly Skin With DIA Mass Spectrometry Reveals New Skin Aging‐Related Proteins,” Aging (Albany NY) 12, no. 13 (2020): 13529–13554. 32602849 10.18632/aging.103461 PMC7377841

[9] S. Kim , K. M. Joo , M. Oh , et al., “Improving Sensitive Skin Diagnosis by Integrating Diagnostic Questionnaires, Lactic Acid Sting Test, and Lipid Profiling,” Journal of Cosmetic Dermatology 24, no. 3 (2025): e70099. 40029145 10.1111/jocd.70099 PMC11875041

[10] W. C. Jiang , J. Wang , H. Zhang , et al., “Seasonal Changes in the Physiological Features of Healthy and Sensitive Skin,” Journal of Cosmetic Dermatology 21, no. 6 (2022): 2581–2589. 34599628 10.1111/jocd.14476

[11] M. M. Ibrahim , L. Chen , J. E. Bond , et al., “Myofibroblasts Contribute to but Are Not Necessary for Wound Contraction,” Laboratory Investigation 95, no. 12 (2015): 1429–1438. 26367489 10.1038/labinvest.2015.116 PMC4861064

[12] L. H. Leng , H. Wang , Y. C. Hu , et al., “LINC02363: A Potential Biomarker for Early Diagnosis and Treatment of Sepsis,” BMC Immunology 26, no. 1 (2025): 23. 40089725 10.1186/s12865-025-00702-x PMC11909972

[13] S. M. Krishna , J. Z. Li , Y. T. Wang , et al., “Kallistatin Limits Abdominal Aortic Aneurysm by Attenuating Generation of Reactive Oxygen Species and Apoptosis,” Scientific Reports 11, no. 1 (2021): 17451. 34465809 10.1038/s41598-021-97042-8 PMC8408144

[14] S. J. Haring , T. D. Humphreys , and M. S. Wold , “A Naturally Occurring Human RPA Subnit Homolog Does Not Support DNA Replication or Cell‐Cycle Progression,” Nucleic Acids Research 38, no. 3 (2010): 846–858. 19942684 10.1093/nar/gkp1062 PMC2817474

[15] V. M. Vassin , M. S. Wold , and J. A. Borowiec , “Replication Protein A (RPA) Phosphorylation Prevents RPA Association With Replication Centers,” Molecular and Cellular Biology 24, no. 5 (2004): 1930–1943. 14966274 10.1128/MCB.24.5.1930-1943.2004 PMC350552

[16] W. Y. Kong , M. T. Longaker , and H. P. Lorenz , “Molecular Cloning and Expression of Keratinocyte Proline‐Rich Protein, a Novel Squamous Epithelial Marker Isolated During Skin Development,” Journal of Biological Chemistry 278, no. 25 (2003): 22781–22786. 12668678 10.1074/jbc.M210488200

[17] H. Suga , T. Oka , M. Sugaya , et al., “Keratinocyte Proline‐Rich Protein Deficiency in Atopic Dermatitis Leads to Barrier Disruption,” Journal of Investigative Dermatology 139, no. 9 (2019): 1867–1875.e7. 30905808 10.1016/j.jid.2019.02.030

[18] P. Krieg , S. Rosenberger , S. D. Juanes , et al., “Aloxe3 Knockout Mice Reveal a Function of Epidermal Lipoxygenase‐3 as Hepoxilin Synthase and Its Pivotal Role in Barrier Formation,” Journal of Investigative Dermatology 133, no. 1 (2013): 172–180. 22832496 10.1038/jid.2012.250

[19] S. C. Konda , A. Biswas , A. Konda , et al., “Ectoderma Dysplasia‐Skin Fragility Syndrome‐Identification of a Novel plakophilin1 (PKP1) Gene Variant Through Whole Exome Sequencing,” Indian Journal of Dermatology, Venereology and Leprology 91, no. 3 (2025): 381–385. 38595014 10.25259/IJDVL_420_2023

[20] Z. Zhang and T. Wang , “YIPF2 Regulates Genome Integrity,” Cell & Bioscience 14, no. 1 (2024): 114. 39238039 10.1186/s13578-024-01300-x PMC11376028

[21] Y. C. Ku , M. H. Lai , C. C. Lo , et al., “DDX3 Participates in Translational Control of Inflammation Induced by Infections and Injuries,” Molecular and Cellular Biology 39, no. 1 (2018): e00285‐18. 30373933 10.1128/MCB.00285-18 PMC6290373

[22] F. M. Rosenberg , R. Wardenaar , A. N. Voorberg , D. C. J. Spierings , and M. L. A. Schuttelaar , “Transcriptional Differences Between Vesicular Hand Eczema and Atopic Dermatitis,” Contact Dermatitis 90, no. 1 (2024): 23–31. 37857578 10.1111/cod.14442

[23] E. Proksch , J. M. Brandner , and J. M. Jensen , “The Skin: An Indispensable Barrier,” Experimental Dermatology 17 (2008): 1063–1072. 19043850 10.1111/j.1600-0625.2008.00786.x

[24] S. Ekanayake‐Mudiyanselage , J. M. Jensen , E. Proksch , H. Aschauer , F. P. Schmook , and J. G. Meingassner , “Expression of Epidermal Keratins and the Cornified Envelope Protein Involucrin Is Influenced by Permeability Barrier Disruption,” Journal of Investigative Dermatology 111, no. 3 (1998): 517–523. 9740250 10.1046/j.1523-1747.1998.00318.x

[25] J. M. Brandner , S. Kief , C. Grund , et al., “Organization and Formation of the Tight Junction System in Human Epidermis and Cultured Keratinocytes,” European Journal of Cell Biology 81 (2002): 253–263. 12067061 10.1078/0171-9335-00244

[26] U. Ohnemus , K. Kohrmeyer , P. Houdek , et al., “Regulation of Epidermal Tight‐Junctions (TJ) During Infection With Exfoliative Toxin‐Negative Staphylococcus Strains,” Journal of Investigative Dermatology 128, no. 4 (2008): 906–916. 17914452 10.1038/sj.jid.5701070

[27] Z. Kopecki , R. J. Ludwig , and A. J. Cowin , “Cytoskeletal Regulation of Inflammation and Its Impact on Skin Blistering Disease Epidermolysis Bullosa Acquistita,” International Journal of Molecular Sciences 17, no. 7 (2016): 1116. 27420054 10.3390/ijms17071116 PMC4964491

[28] A. I. Ivanov , C. A. Parkos , and A. Nusrat , “Cytoskeletal Regulation of Epithelial Barrier Function During Inflammation,” American Journal of Pathology 177, no. 2 (2010): 512–524. 20581053 10.2353/ajpath.2010.100168 PMC2913378

[29] K. D. Sumigray and T. Lechler , “Cell Adhesion in Epidermal Development and Barrier Formation,” Current Topics in Developmental Biology 112 (2015): 383–414. 25733147 10.1016/bs.ctdb.2014.11.027 PMC4737682

[30] S. Freeman and S. Sonthalia , “Histology, Keratohyalin Granules,” in StatPearls [Internet] (StatPearls publishing, 2023). 30725734

[31] E. V. Kalinina , N. N. Chernov , and M. D. Novichkova , “Role of Glutathione, Glutathione Transferase, and Glutaredoxin in Regulation of Redox‐Dependent Processes,” Biochemistry 79, no. 13 (2014): 1562–1583. 25749165 10.1134/S0006297914130082

[32] A. Sreedhar , L. Aguilera‐Auirre , and K. K. Singh , “Mitochondria in Skin Health Aging, and Disease,” Cell Death & Disease 11, no. 6 (2020): 444. 32518230 10.1038/s41419-020-2649-z PMC7283348

[33] G. Mcknight , J. Shah , and R. Hargest , “Physiology of the Skin,” Surgery (Oxford) 40, no. 1 (2022): 8–12.

[34] S. Jeayeng , J. Kwanthongdee , R. Jittreeprasert , et al., “Natural Products as Promising Therapeutics for Fine Particulate Matter‐Induced Skin Damage: A Review of Pre‐Clinical Studies on Skin Inflammation and Barrier Dysfunction,” PeerJ 13 (2025): e19316. 40313388 10.7717/peerj.19316 PMC12045276

[35] S. Dunaway , R. Odin , L. L. Zhou , et al., “Natural Antioxidants: Multiple Mechanisms to Protect Skin From Solar Radiation,” Font Pharmacol 9 (2018): 392. 10.3389/fphar.2018.00392 PMC5928335 29740318

[36] S. Dong , D. M. Li , and D. M. Shi , “Skin Barrier‐Inflammatory Pathway Is a Driver of the Psoriasis‐Atopic Dermatitis Transition,” Frontiers in Medicine 11 (2024): 1335551. 38606161 10.3389/fmed.2024.1335551 PMC11007107

[37] J. W. Deng , V. Parthasarathy , M. Marani , et al., “Extracellular Matrix and Dermal Nerve Growth Factor Dysregulation in Prurigo Nodularis Compared to Atopic Dermatitis,” Frontiers in Medicine 9 (2022): 1022889. 36619628 10.3389/fmed.2022.1022889 PMC9810753

[38] A. Hodeib , Z. A. El‐Samad , H. Hanafy , et al., “Nerve Growth Factor, Neuropeptides and Cutaneous Nerves in Atopic Dermatitis,” Indian Journal of Dermatology 55, no. 2 (2010): 135–139. 20606880 10.4103/0019-5154.62735 PMC2887515

[39] O. Mahmoud , O. Oladipo , R. Mahmoud , et al., “Itch: From the Skin to the Brain‐Peripheral and Central Neural Sensitization in Chronic Itch,” Frontiers in Molecular Neuroscience 16 (20223): 1272230. 10.3389/fnmol.2023.1272230 PMC10577434 37849619

[40] S. Dhall , D. S. Wijesinghe , Z. A. Karim , et al., “Arachidonic Acid‐Derived Signaling Lipids and Functions in Impaired Healing,” Wound Repair and Regeneration 23, no. 5 (2015): 644–656. 26135854 10.1111/wrr.12337 PMC4837701

[41] C. Manosalva , P. Alarcon , K. Gonzalez , et al., “Free Fatty Acid Receptor 1 Signaliing Contributes to Migration, MMP‐9 Activity, and Expression of IL‐8 Induced by Linoleic Acid in HaCaT Cells,” Frontiers in Pharmacology 11 (2020): 595. 32431615 10.3389/fphar.2020.00595 PMC7216565

[42] D. Staumont‐salle , G. Abboud , C. Brenuchon , et al., “Peroxisome Proliferator‐Activated Receptor Alpha Regulates Skin Inflammation and Humoral Response in Atopic Dermatitis,” Journal of Allergy and Clinical Immunology 121, no. 4 (2008): 962–968. 18249437 10.1016/j.jaci.2007.12.1165

[43] Q. H. Yuan , F. Xie , W. Huang , et al., “The Review of Alpha‐Linolenic Acid: Sources, Metabolism, and Pharmacology,” Phytotherapy Research 36, no. 1 (2022): 164–188. 34553434 10.1002/ptr.7295

[44] K. Ono , C. T. Viet , Y. Ye , et al., “Cutaneous Pigmentation Modulates Skin Sensitivity via Tyrosinase‐Dependent Dopaminergic Signalling,” Scientific Reports 7, no. 1 (2017): 9181. 28835637 10.1038/s41598-017-09682-4 PMC5569050

