# Correction to “A Systemic Pathophysiological View of Sensitive Skin Revealed by Proteomics: Beyond Barrier and Inflammation”

**DOI:** 10.1111/jocd.70723

**Published:** 2026-02-09

L. Guihua, D. Shan, Z. Xinqing, L. Yunha, and X. Zhi, “A Systemic Pathophysiological View of Sensitive Skin Revealed by Proteomics: Beyond Barrier and Inflammation,” *Journal of Cosmetic Dermatology* 25, no. 1 (2026): e70649, https://doi.org/10.1111/jocd.70649.

In the originally published version of this article, errors were identified in the reference citations.

Specifically, in Section 2.5 (AMOREPACIFIC Skin Sensitivity Questionnaire), the reference citation highlighted in the published article was incorrect and should have cited References 4 and 9. In addition, in Section 3.1 (Differential Protein Expression Analysis Between SS and NS), the reference citation highlighted in the published article was incorrect and should have cited Reference 13.

The detailed revisions are as follows:

**2.5 AMOREPACIFIC Skin Sensitivity Questionnaire**

The classification of participants into Sensitive Skin (SS) and Non‐Sensitive Skin (NS) groups was determined using a multi‐step approach. Initially, all participants completed a validated self‐assessment questionnaire for sensitive skin [4, 9]. The final composite sensitive skin score was calculated as (Category A sum × 2) + (Category B sum × 3). Participants with a total score ≥ 10 were preliminarily classified as questionnaire SS, whereas those with a score < 10 were classified as questionnaire NS (Table 1).

**3.1 Differential Protein Expression Analysis Between SS and NS**

Using DIA‐MS (Data‐Independent Acquisition Mass Spectrometry), a total of 4373 proteins were identified from stratum corneum samples. Applying criteria of fold change > 1.5 or < 0.67 and *p* < 0.05, 583 proteins were differentially expressed between the SS and NS, including 336 upregulated and 247 downregulated proteins (Figure 1). Among the top upregulated proteins in SS were ACTG1 (barrier‐related [11]), DYNLT1 and SERPINA4 (inflammation [12] and oxidative stress‐related [13]), and RPA2 (DNA damage and repair‐related [14, 15]) (Table 2). The top downregulated proteins included KPRP, ALOXE3, and PKP1 (barrier‐related [16–19]), YIPF2 (DNA damage response‐related [20]), GNB2 (inflammation‐related [21]), and KRT9 (epidermal differentiation‐related [22]) (Table 3).

These errors occurred during manuscript preparation and do not affect the results, interpretation, or conclusions of the study.

We apologize for this error.

